# Will Posttranslational Modifications of Brain Proteins Provide Novel Serological Markers for Dementias?

**DOI:** 10.1155/2012/209409

**Published:** 2012-06-21

**Authors:** Y. Wang, M. G. Sørensen, Q. Zheng, C. Zhang, M. A. Karsdal, K. Henriksen

**Affiliations:** ^1^Department of Biomarker Development, Nordic Bioscience A/S, Beijing 102206, China; ^2^Neurodegenerative Diseases, Nordic Bioscience A/S, Herlev Hovedgade 207, 2730 Herlev, Denmark

## Abstract

Drug development for dementias is significantly hampered by the lack of easily accessible biomarkers. Fluid biomarkers of dementias provide indications of disease stage, but have little prognostic value, cannot detect early pathological changes, and can only be measured in CSF (cerebrospinal fluid) which significantly limits their applicability. In contrast, imaging based biomarkers can provide indications of probability of disease progression, yet are limited in applicability due to cost, radiation and radio-tracers. These aspects highlight the need for other approaches to the development of biomarkers of dementia, which should focus on not only providing information about pathological changes, but also on being measured easily and reproducibly. For other diseases, focus on development of assays monitoring highly specific protease-generated cleavage fragments of proteins has provided assays, which in serum or plasma have the ability to predict early pathological changes. Proteolytic processing of brain proteins, such as tau, APP, and **α**-synuclein, is a key pathological event in dementias. Here, we speculate that aiming biomarker development for dementias at detecting small brain protein degradation fragments of generated by brain-derived proteases specifically in blood samples could lead to the development of novel markers of disease progression, stage and importantly of treatment efficacy.

## 1. Introduction 

Dementias are a group of disorders characterized by declining cognitive function, usually with increasing prevalence as a function of aging. Several types of dementia exist, with Alzheimer's disease (AD) being the most prominent, followed by dementia with Lewy bodies (DLB), frontotemporal dementia (FTD) also known as frontotemporal lobar degeneration (FTLD), corticobasal degeneration (CBD), and vascular dementia (VaD) [1]. According to the World Alzheimer Report 2009, it has been estimated that 36 million people worldwide are affected by dementia, with numbers doubling every 20 years [2]. Women are overrepresented in terms of incidence of dementias, with 3.4 of 5.4 million Americans living with Alzheimer being females [3]. 

By the time dementia can be clinically diagnosed, neuropathologic features are already extensive in some regions of brain, and hence some of the pathological changes appear irreversible. Therefore, the general consensus is that dementia should be diagnosed as early as possible, and thereby improving the possibility for intervention [4–7].

Genetic studies have shown that individuals with mutations in the genes for *APP*, *PSEN1*, or *PSEN2* genes are predisposed for early onset AD; however, only a very low percentage of AD patients have these mutations [3]. In contrast, between 40 and 80% of AD cases have the APOE *ε*4 allele [8, 9]. Having this allele either as a heterozygotic or homozygotic allele raises the probability for Alzheimer's by three and 15 times respectively [3]. 

Mutations in several other genes are known to predispose the carriers for different forms of dementia, and these include mutations in the *MAPT* gene, which encodes tau, and these mutations can lead FTD, CBD, and other forms of dementia [10]. In addition, mutations in the *PGRN* gene encoding progranulin can cause FTD [11, 12]. Several other gene mutations are known to predispose carriers for different forms of dementia; however, these are beyond the scope of this paper, and we refer to [10].

Besides aging, risk factors for dementia are hypercholesterolaemia, hypertension, atherosclerosis, coronary heart disease, head injuries, smoking, obesity, and diabetes; however, whether they are causal factors or not is still undergoing investigation [3, 13, 14].

At present, a diagnosis of dementia can be reached through neuropsychological testing, imaging-based analysis, and through the use of CSF biomarkers [15, 16]. However, while a positive diagnosis of AD can be obtained with a rather large certainty, the picture is more complicated for other dementias, where misdiagnosis and mixed pathologies are complicating factors [17]. Although imaging-based analysis can allow early diagnosis as well as provide some prognostic value, the limitations are still many [15]. Furthermore, for several of the dementias, it is well known that the pathological changes start appearing markedly earlier than the cognitive decline [4–7, 15], and hence there is a substantial need for a novel approach to biomarkers of dementia. 

With respect to treatment of dementias, the presently available treatment possibilities only provide symptomatic relief or slow the cognitive decline moderately, but temporarily, and following the reduction in progression neuronal degradation accelerates again [3, 15, 18]. Despite failures in drug development for dementias, including prominent AD phase III trial failure semagacestat [19], there are still numerous clinical trials ongoing. 

A major issue in the development of drugs for dementias is the lack of biomarkers allowing the selection of the cohort, that is, patients who have not yet reached a point-of-no-return and will show disease progression during the study. Secondly, biomarkers of treatment efficacy are lacking, although this could also be a result of the lack of useful biomarkers for inclusion of patients in the studies [15, 20–25]. 

In this paper, we ask the following question: is it possible to develop a biomarker system allowing the detection of neuronal pathology in the circulation by focusing on protease-generated protein fragments? This would allow easier and more frequent sampling and analysis and potentially promote earlier diagnosis and prognosis and thereby allow monitoring of treatment efficacy. 

## 2. Pathological Proteins in Dementia

Converging lines of investigation have revealed potential common pathogenic mechanisms underlying many diverse dementias [10, 26], and, interestingly, dementias are in general characterized by faulty protein processing mechanisms, resulting in accumulation of pathological forms of brain proteins. The pathological processing is a crucial step towards induction of neuronal apoptosis, and hence monitoring these processes would be of great interest as biomarkers of neuronal status. 

Most dementias are characterized by similar molecular mechanisms, including protein cleavage, protein aggregation, and inclusion body formation in selected regions of brain and thereby the formation of numerous alterations in the brain proteins resulting in the formation of posttranslational modifications (PTMs), also referred to as neoepitopes, that is, novel sites not previously presented in the body [27–29].

In AD, there are two well-described processes, namely, (1) the formation of A*β* senile plaques, through an imbalance in the processing of APP (see Table 1), and APP processing appears to be an initiating factor for AD [29], (2) the formation of neurofibrillary tangles (NFTs) through pathological processing of tau involving phosphorylations and protease cleavage, and subsequent aggregation [28], a process that appears to be directly involved in triggering neuronal death [30, 31]. Interestingly, recent data indicate that A*β* is involved in inducing cleavage of tau and subsequent neuronal apoptosis [30, 32], thus providing some mechanistic insight into the pathology of AD. 

Dementia with Lewy bodies on the other hand is characterized not only by having NFTs but also inclusions consisting of aggregations of pathologically processed *α*-synuclein (Lewy bodies), while the presence of amyloid plaques is very limited [33–35].

In FTD, accumulations of pathologically processed proteins are also observed, and in these cases the proteins TAR DNA-binding protein 43 (TDP-43) and FUsed in Sarcoma (FUS) are highly relevant and are known to undergo several pathological changes, which all contribute to neuronal toxicity [37, 38]. The accumulations of these proteins are an important part of the subclassification of FTD into more specific categories [39].

Interestingly, CBD and VaD are primarily tauopathies and are characterized by accumulations of NFTs in the neurons, and while other pathological changes are known to occur, these do not appear to include other protein aggregations, although some AD-like traits are observed [40, 41].

These mechanisms are interrelated in complex vicious cycles which lead to cell dysfunction and death. However, in relation to development of novel biomarkers, the processing steps the individual proteins undergo are of great interest [27]. 

## 3. Presently Available Biomarkers and Why Alternatives Are Needed

Biomarkers for the different forms of dementia, and especially AD, have been investigated for a long time, and hence numerous biomarkers exist. These are based on imaging and CSF-based technologies, which can detect and monitor dementia, but they are limited by aspects such as the need for lumbar puncture, use of radiotracers, and lack of predictive value in terms of both disease progression, but also response to treatments in development [15, 43].

As the cognitive decline only can be observed several years after the onset of neuropathological changes, cognitive tests are mainly useful at a diagnostic level [15, 44]. The most commonly used imaging modality is MRI, which allows assessment of both static and dynamic parameters in the brain [15]. These techniques have predictive value and can measure many of the desired changes in the brain, such as cortical atrophy, neuronal integrity, and more [15]. 

In addition, methods such as FDG-PET and ^18^C-PIB scans also provide essential information about brain status and progression of disease [15, 43, 45]; however, they are significantly limited by the number of scans allowed due to radiation and costs as well as patient discomfort/unruliness associated with being placed in the scanner [16, 46, 47].

CSF-derived biomarkers for dementias, include A*β*(1–42), total tau protein, and hyperphosphorylated tau, and these protein species have well-established patterns in dementias [21, 48–51]. However, while they are of use for diagnosis and segregation of disease, their use is still somewhat limited by the need for lumbar puncture, a procedure perceived as unpleasant and risky. Lumbar puncture cannot be performed as often as desired when monitoring treatment efficacy [51, 52]. Furthermore, the CSF biomarkers have limited prognostic value [15].

**Figure 1:**
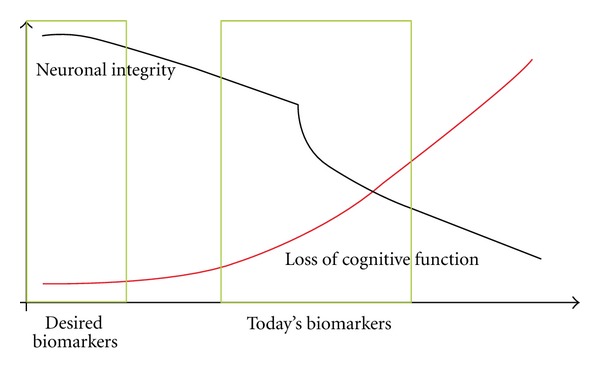
Schematic illustration of the alterations in neuronal integrity (black line) and subsequent loss of cognitive function (red line). The green boxes indicate at what level the presently available biomarkers have diagnostic/prognostic value, that is, once cognitive decline has begun, and at which level it is desired to be able to provide a prognosis/diagnosis, that is, biomarkers monitoring very early changes in neuronal integrity.

As illustrated in Figure 1, novel biomarkers of dementias should focus on detection of very early stages, which will allow treatment at the right time and assist in selection of patients at risk for progression [15, 27]. In addition, biomarkers monitoring treatment efficacy, especially at the level of neuronal integrity would be of great use [15, 27].

In summary, early diagnosis before the cognitive decline accelerates, selection of those who will show progression of disease, and monitoring of treatment efficacy, all those aspects ideally require the identification of effective biomarkers. These biomarkers should preferably be monitored in serum and/or plasma, as this would allow a more frequent sampling and hence a more detailed understanding of both pathology and effects of treatments.

## 4. Present Fluid Biomarkers for AD and Their Classification and Utility

Correction application of biomarkers is facilitated by a useful classification, and, within the field of osteoarthritis, five useful categories have been proposed. These categories are referred to as the BIPED criteria and they introduce a simple and useful way of separating biomarkers (Table 2 and [36]). 

As illustrated in Table 3, the presently available fluid biomarkers for dementias primarily belong in the burden of disease and diagnostic categories [48], and while these are highly useful in terms of strengthening diagnosis, can provide a modest segregation into different dementia types, and are applied across this field, and fluid biomarkers of treatment efficacy, and importantly with predictive value, are still lacking to a great extent [15, 42, 53]. One major reason for these limitations is most likely the need for collection of CSF, which requires a lumbar puncture and which cannot be performed frequently [16]. 

Several studies have examined the potential of measuring A*β* and tau in plasma/serum samples, and A*β* is present in plasma but the levels are not related to pathology [54–56], a finding likely explained by the large level of A*β* bound to immunoglobulins in plasma [57]. On the other hand, plasma A*β* appears to respond to *γ*-secretase inhibitor treatment and hence appears to be relevant as an efficacy marker for drugs affecting the *γ*-secretase [58]. In addition, a study has monitored switches in isoforms of APP in plasma as a function of AD, and it appears that there is a switch towards lower molecular weight isoforms in AD [59]. 

Serum/plasma tau levels have been explored extensively, but tau is virtually undetectable in MCI and/or AD [60]; however, serum tau levels are elevated in a series of other pathologies with a markedly different pathology, such as ischemic stroke [61], Creutzfeldt-Jacob's disease [60], and traumatic brain injury [62]. For hyperphosphorylated tau, there are no studies clearly showing any relevance of this marker in serum/plasma [15, 63].

Hence, monitoring in serum/plasma at present is limited to experimental markers, such as the 18 peptide profile described which initially was thought to be useful for segregating different dementias [64], or the blood-based algorithm by O'Bryant and colleagues [65]. However, underscoring the complex nature of monitoring brain pathologies in blood specimens is a recent study finding that the 18 peptide profile cannot segregate dementias and only have limited diagnostic value in another cohort [66]. These points clearly illustrate the need for novel approaches for identification of fluid biomarkers for dementias.

## 5. Critical Considerations for the Design of Serum Biomarkers for Dementia

A major issue in relation to serum detection of brain-derived proteins is the blood-brain barrier (BBB), which does not allow large proteins to cross. The (BBB) exists between the peripheral circulation and brain, and its primary function is to protect the brain from potentially harmful substances present in blood [67, 68]. However, in addition to reducing entry into the brain, the BBB also reduces exit of molecules from the brain [68, 69], a function which has complicated the biomarker development process significantly and which is the main reason for the lack of useful serum biomarkers for dementias.

Of importance is that CSF is absorbed into blood every day, and some exchanges of peptides occur, meaning that a protein fragment of sufficiently small size may have the possibility to pass BBB potentially allowing detection in serum or plasma [16]. An example of this is that A*β* is present in plasma, but is bound to immunoglobulins, and thus cannot be reliably used for diagnosis [57].

**Figure 2:**
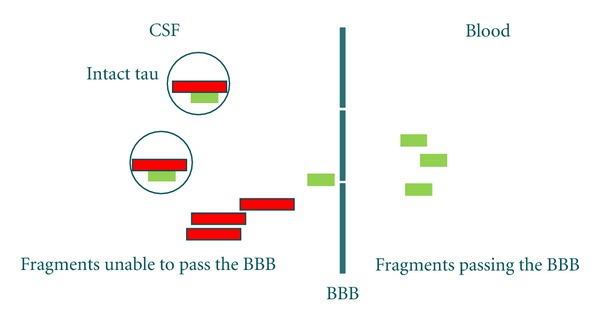
Schematic illustration of proteins present in the CSF, and the possibility that small fragments can cross.

All of the dementias are characterized by pathological protein processing, of which fragmentation and other posttranslational modifications (PTMs) appear to be key pathological events [31, 70–72]. With these aspects in mind, we speculate that protease-mediated cleavage of brain proteins will lead to the generation of small fragments which can be released into the serum, and which in serum will represent neoepitopes of potential use for serum detection (Figure 2).

## 6. Neuronal Proteins and Proteases of Interest for Development of Dementia Biomarkers

Dementias are characterized by aberrant protein processing, and the processed proteins have for a long time been explored and used as biomarkers for dementias [15, 42]. These include A*β*(1–42) and hyperphosphorylated tau, both of which represent pathological processes ongoing in the brain; however, data generated using these markers is only meaningful in CSF as the free levels of these present in serum are extremely low [15, 57]. 

When examining the forms of dementia, several proteins and proteases are known to show alterations [28, 29]. 

Classical examples of pathological processing steps in neuronal proteins include *γ*-secretase cleaved amyloid precursor protein (APP) to generate the 1–42 amino acid polypeptide (A*β*), which forms toxic oligomers and eventually deposits as plaques [28]. 

Microtubule-associated protein tau undergoes several posttranslational modifications, and recent data have indicated that the caspase cleavage at the C-terminal is a key early event occurring, which appears to occur only in the absence of phosphorylation and which causes aggregation into the neurofibrillary tangles [30, 73–75] (Table 2). 

In addition to tau and APP processing, processing of *α*-synuclein and TDP-43 and several other proteins with high specificity for the brain are known to be processes by different classes of proteases during pathological events, and these known processing steps related to dementia are listed in Table 4.

We speculate that in addition to these truncations several others will take place and that selective searching for fragments of the proteins of interest in serum/plasma could provide useful biomarkers of neuronal pathologies. 

## 7. The Potential of Posttranslational ****Modifications (PTMs) as Biomarkers

Importantly, several different posttranslational modifications (PTMs) exist, and these include the aforementioned generation of novel protease cleavage sites, isomerizations, crosslinks, phosphorylations, nitrosylations, glycosylations, glycations, hydroxylations, and more [27].

The dementia field has for many years taken advantage of the fact that pathologies introduce PTMs in proteins, and this approach has been duplicated across many research fields [27].

Within the dementia field, the best characterized PTM-based biomarkers are the A*β*(1–42) fragment and hyperphosphorylated tau, but of which, as mentioned previously, are used diagnostically for AD [15, 42]. However, as described earlier, despite the application of PTM approach, there are still several limitations to the fluid biomarkers [16]. 

Hence, other peripheral biomarkers, such as serological biomarkers which could provide us noninvasive, inexpensive, convenient, and frequent samples, are intensely sought [16]. One promising approach towards this goal is to focus on the size of fragments generated by protease cleavage and then work selectively on identifying small fragments of proteins in circulation, and these could then be explored for their biomarker potential. Interestingly, recent studies have highlighted the C-terminal truncation of tau causing generation of the protein species referred to as tau-C3 as a key initiator of tau processing ultimately causing NFTs and death [31, 72–76]; however, whether the tau-C3 can be measured in fluids such as CSF or even serum has not yet been explored. 

If successful, this approach could be followed by searching for other PTMs present in the identified fragments, and as both phosphorylation and glycations are known to occur in dementia pathologies these are candidates of interest [77–79].

## 8. An Example of What a PTM-Based Biomarker Can Do

A*β* is one of the most used PTM/neoepitope biomarkers, and as described previously in this paper, it is highly useful for diagnosis of AD. However, as also described there are also significant limitations to its use [15, 42].

There is in particular one PTM biomarker in serum, which has been used extensively, and this is the bone resorption marker *β*-CTX-I, and the use of this marker has illustrated many of the benefits and a few of the challenges of this class of biomarkers [80]. 

In bone, the ECM consists of 90% type I collagen, and this matrix is degraded by the bone resorbing osteoclast [81]. The osteoclasts degrade type I collagen using the cysteine proteinase cathepsin K, and this has been shown to lead to the generation of the CTX fragment (^1207^EKAHDDGR^1214^) [82–84]. 

The CTX fragment hence contains a cathepsin K cleavage site as its primary PTM; however, in addition, it is a dipeptide linked together via a lysine crosslink adding another PTM [27, 85]. Finally, it contains a DG amino acid sequence, and this site undergoes isomerization with time, and the *β*-CTX-I system measures the isomerized, hence aged, form, thereby including one more PTM in this system [85]. 

Studies of b-CTX have shown that it is elevated in postmenopausal women, it has predictive value for osteoporotic fractures, it not only responds to treatment but also predicts effect of the antiresorptive treatment on BMD [80], and hence it fits into all the BIPED categories [86].

In relation to the issues of *β*-CTX-I, measurements of *β*-CTX-I in studies have highlighted that it is very rapidly removed from circulation, which allows the measurement of changes in levels within less than one hour after treatment in human studies [87–89]. Furthermore, it exhibits a pronounced diurnal variation, which correlates directly to observation of osteoclast activity and which appears to be controlled almost completely by food intake [87–89]. The sensitivity of CTX-I to food intake/diurnal variation is often seen as a limitation; however, on the other hand, when knowing how to handle this variation, CTX-I is a highly sensitive and rapidly responsive biomarker, and, therefore, this unique triple PTM biomarker is among the most used biomarkers in both in vitro preclinical and clinical studies within the bone field as well as in studies of the bone safety of other drugs, that is, glitazones and Serotonin Reuptake Inhibitors [90, 91]. 

We speculate that utilizing the knowledge about PTMs/neoepitopes from the bone field may provide possibilities for biomarkers development within the dementia fields, although this still remains to be demonstrated conclusively.

## 9. Conclusions and Challenges

While there are imaging- and CSF-based technologies for the diagnosis of dementias, there is still a large unmet need for novel markers, especially markers which can predict disease progression and treatment response, and preferably in serum or plasma as this allows more frequent sampling at a markedly lower risk for complications.

The major hurdle in the development of serum biomarkers for dementias is the limited passage of proteins or fragments through the BBB, as underlined by the difficulties in detecting intact and phosphorylated tau in serum samples [68]. This is where the protease-generated protein fragments, often referred to as neoepitopes, are of great interest, as these provide the possibility for getting a protein fragment of sufficiently small size to allow crossing of the BBB, but also with pathological relevance and a high level of specificity due to the possibility of combining specific brain proteins with specific brain proteases. Interestingly, there are already studies showing that specific pathological processing of tau results in the generation of a highly interesting fragment [31, 71–75], and although it still is unclear whether this fragment can be utilized as a serum marker, this is a promising finding. 

If successful, this approach could also allow differential diagnosis of the different dementias, as they are characterized by different enzymatic processing steps, as well as a series of other PTMs, all which can be explored in the context of biomarker development, and thus potentially result in a biomarker panel with the ability to clearly separate different forms based on their protein degradation profile. An example could be a caspase-generated tau fragment specific for AD versus a calpain-generated *α*-synuclein fragment to separate AD from DLB [48].

However, the probability of success is to a large extent defined by the ability to identify small brain-derived fragments in serum, and even if these are identified they are likely to be present at low concentrations, which will increase the demand for a highly sensitive and highly specific detection system. Furthermore, as illustrated by the *β*-CTX-I example, numerous parameters need to be investigated in detail in order to validate the relevance of a potential marker [80], and whether this approach or other approaches, that is, mass spectrometry-based analyses of CSF and/or plasma samples, will end up providing a fluid biomarker with a broader application remains to be seen. 

Another interesting aspect in relation to dementias is the possibility of monitoring comorbidities, such as diabetes and/or cardiovascular disease [92, 93]. As biomarkers of these pathologies exist, combining them with novel dementia biomarkers could provide additional strength to the analysis of the patients, and thus ultimately help segregation of patients for clinical trials, for the right type of treatment, and so forth. 

In summary, although the dementia field has been working on biomarker development for several years, there is still a large area that has not been explored, namely, the small degradation fragments in serum. Based on the success of this approach within other diseases where biomarkers have been lacking, we are enthusiastic about this possibility, despite the numerous hurdles that will need crossing. 

## Figures and Tables

**Table 1:** Relevant proteins for each dementia (see text for references).

Type of dementia	Relevant protein
Alzheimer's disease (AD)	Tau, A*β*
Dementia with Lewy bodies (DLB)	*α*-Synuclein, tau
Frontotemporal dementia (FTD)	Tau, TDP-43, FUS
Vascular dementia (VaD)	Tau
Corticobasal degeneration (CBD)	Tau

**Table 2:** BIPED classification adopted from [36].

Burden of disease:	Burden-of-disease markers assess the severity or extent of disease
Investigative:	A marker which does not have a clear-cut pathological relevance, but is used exploratively
Prognostic:	The key feature of a prognostic marker is the ability to predict the future onset of disease
Efficacy of intervention:	An efficacy-of-intervention biomarker provides information about the efficacy of treatment or those at high risk for its development
Diagnostic:	Diagnostic markers are defined by the ability to classify individuals as either having or not having a disease

**Table 3:** Present fluid biomarkers for dementias from [15, 33, 42].

Analysis sample	Biomarker	BIPED classification	Relationship with pathology
CSF	t-tau	B,D	Increased in AD, indicates the neuronal degeneration
	p-tau	B,D	Increased in AD, reflects the formation of tangles
	A*β*42	B,D	Reduced in the onset stage of AD, it remains unchanged after onset of AD
	A*β* oligomers	B,D	Increased in AD
	APPs-a	B,D	Soluble APPa is decreased in AD
	APPs-*β*	B,D	APPs-*β* is a product of APP cleavage by BACE-1; it cannot discriminate normal from AD
	APLI*β*	B,D	Fragments generated by *β*- and *r*-secretase are increased in AD
	*α*-Synuclein	B,D	There is an inverse relationship between severity of disease and *α*-synuclein, it increases rapidly after neuron death in DLB
	BACE-1	B,D	Increased activity in MCI but not AD

Plasma	A*β*40,42	E	Plasma A*β* is in large amount bound to plasma protein, it cannot discriminate normal from AD, but may have a role as an efficacy marker

**Table 4:** Proteins, proteases, and the consequences in relation to dementia.

Protein	Normal function	Protease	Alteration and consequence	Disease	Reference
APP	Lipid metabolism, axonal transport??	*α*,*β*,*γ*-Secretases ADAMs MMPs	Fragmentation, generation of A*β*, formation of amyloid plaques	AD	[28, 94, 95]
tau	Microtubule stabilizing protein	Caspase Calpain	C-terminal truncation in AD and aggregation causing NFTs	AD	[28, 73–75]
*α*-Synuclein	Molecular chaperone	MMPs calpain cathepsins	Truncation and aggregation leading to Lewy bodies	DLB	[34, 35, 96, 97]
TDP-43	Transcription and splicing regulation, apoptosis, cell division, and stabilisation of messenger RNA	Caspase?	C-terminal truncation, aggregation formation of Lewy bodies	FTLD-TDPAD	[37, 39, 98, 99]
FUS	Transcription factor	??	??	FTLD-FUS	[39]
GFAP	Neurofilament	Caspase	Truncation and neuronal death	Alexander disease	[100]

## References

[1] Karantzoulis S, Galvin JE (2011). Distinguishing Alzheimer’s disease from other major forms of dementia. *Expert Review of Neurotherapeutics*.

[2] World Alzheimer Report 2009

[3] Blennow K, de Leon MJ, Zetterberg H (2006). Alzheimer’s disease. *The Lancet*.

[4] Forstl H, Kurz A (1999). Clinical features of Alzheimer’s disease. *European Archives of Psychiatry and Clinical Neuroscience*.

[5] http://www.nia.nih.gov/alzheimers/topics/symptoms.

[6] Molsa PK, Marttila RJ, Rinne UK (1986). Survival and cause of death in Alzheimer’s disease and multi-infarct dementia. *Acta Neurologica Scandinavica*.

[7] Cairns NJ, Bigio EH, Mackenzie IRA (2007). Neuropathologic diagnostic and nosologic criteria for frontotemporal lobar degeneration: consensus of the consortium for frontotemporal lobar degeneration. *Acta Neuropathologica*.

[8] Mahley RW, Weisgraber KH, Huang Y (2006). Apolipoprotein E4: a causative factor and therapeutic target in neuropathology, including Alzheimer’s disease. *Proceedings of the National Academy of Sciences of the United States of America*.

[9] Mahley RW, Huang Y (2009). Alzheimer disease: multiple causes, multiple effects of apolipoprotein E4, and multiple therapeutic approaches. *Annals of Neurology*.

[10] Jellinger KA (2009). Recent advances in our understanding of neurodegeneration. *Journal of Neural Transmission*.

[11] Yu CE, Bird TD, Bekris LM (2010). The spectrum of mutations in progranulin: a collaborative study screening 545 cases of neurodegeneration. *Archives of Neurology*.

[12] van Swieten JC, Heutink P (2008). Mutations in progranulin (GRN) within the spectrum of clinical and pathological phenotypes of frontotemporal dementia. *The Lancet Neurology*.

[13] Barnes DE, Yaffe K (2011). The projected effect of risk factor reduction on Alzheimer’s disease prevalence. *The Lancet Neurology*.

[14] McKhann GM, Albert MS, Grossman M, Miller B, Dickson D, Trojanowski JQ (2001). Clinical and pathological diagnosis of frontotemporal dementia: report of the work group on frontotemporal dementia and Pick’s disease. *Archives of Neurology*.

[15] Cummings JL (2011). Biomarkers in Alzheimer’s disease drug development. *Alzheimer’s and Dementia*.

[16] Patel S, Shah RJ, Coleman P, Sabbagh M (2011). Potential peripheral biomarkers for the diagnosis of Alzheimer’s disease. *International Journal of Alzheimer’s Disease*.

[17] Josephs KA, Petersen RC, Knopman DS (2006). Clinicopathologic analysis of frontotemporal and corticobasal degenerations and PSP. *Neurology*.

[18] Karakaya T, Fusser F, Prvulovic D, Hampel H (2012). Treatment options for tauopathies. *Current Treatment Options in Neurology*.

[20] Thambisetty M, Lovestone S (2010). Blood-based biomarkers of Alzheimers disease: challenging but feasible. *Biomarkers in Medicine*.

[21] Hoglund K, Hansson O, Buchhave P (2008). Prediction of Alzheimer’s disease using a cerebrospinal fluid pattern of C-terminally truncated *β*-amyloid peptides. *Neurodegenerative Diseases*.

[22] Zetterberg H, Andreasson U, Hansson O (2008). Elevated cerebrospinal fluid BACE1 activity in incipient Alzheimer disease. *Archives of Neurology*.

[23] Zetterberg H (2008). Biomarkers reflecting different facets of Alzheimer’s disease. *European Journal of Neurology*.

[24] Schroeter ML, Stein T, Maslowski N, Neumann J (2009). Neural correlates of Alzheimer’s disease and mild cognitive impairment: a systematic and quantitative meta-analysis involving 1351 patients. *NeuroImage*.

[25] Takeda S, Sato N, Rakugi H, Morishita R (2010). Plasma *β*-amyloid as potential biomarker of Alzheimer disease: possibility of diagnostic tool for Alzheimer disease. *Molecular BioSystems*.

[26] Crews L, Masliah E (2010). Molecular mechanisms of neurodegeneration in Alzheimer’s disease. *Human Molecular Genetics*.

[27] Karsdal MA, Henriksen K, Leeming DJ, Woodworth T, Vassiliadis E, Bay-Jensen AC (2010). Novel combinations of post-translational modification (PTM) neo-epitopes provide tissue-specific biochemical markers—are they the cause or the consequence of the disease?. *Clinical Biochemistry*.

[28] De Strooper B (2010). Proteases and proteolysis in Alzheimer disease: a multifactorial view on the disease process. *Physiological Reviews*.

[29] De Strooper B, Annaert W (2000). Proteolytic processing and cell biological functions of the amyloid precursor protein. *Journal of Cell Science*.

[30] Reifert J, Hartung-Cranston D, Feinstein SC (2011). Amyloid *β*-mediated cell death of cultured hippocampal neurons reveals extensive tau fragmentation without increased full-length Tau phosphorylation. *The Journal of Biological Chemistry*.

[31] Gamblin TC, Chen F, Zambrano A (2003). Caspase cleavage of tau: linking amyloid and neurofibrillary tangles in Alzheimer’s disease. *Proceedings of the National Academy of Sciences of the United States of America*.

[32] Guillozet-Bongaarts AL, Garcia-Sierra F, Reynolds MR (2005). Tau truncation during neurofibrillary tangle evolution in Alzheimer’s disease. *Neurobiology of Aging*.

[33] Noguchi-Shinohara M, Tokuda T, Yoshita M (2009). CSF *α*-synuclein levels in dementia with Lewy bodies and Alzheimer’s disease. *Brain Research*.

[34] Sung JY, Park SM, Lee CH (2005). Proteolytic cleavage of extracellular secreted *α*-synuclein via matrix metalloproteinases. *The Journal of Biological Chemistry*.

[35] Dufty BM, Warner LR, Hou ST (2007). Calpain-cleavage of *α*-synuclein: connecting proteolytic processing to disease-linked aggregation. *American Journal of Pathology*.

[36] Bauer DC, Hunter DJ, Abramson SB (2006). Classification of osteoarthritis biomarkers: a proposed approach. *Osteoarthritis and Cartilage*.

[37] Yang C, Tan W, Whittle C (2010). The C-terminal TDP-43 fragments have a high aggregation propensity and harm neurons by a dominant-negative mechanism. *PLoS ONE*.

[38] Ito D, Suzuki N (2011). Conjoint pathologic cascades mediated by ALS/FTLD-U linked RNA-binding proteins TDP-43 and FUS. *Neurology*.

[39] Mackenzie IRA, Rademakers R, Neumann M (2010). TDP-43 and FUS in amyotrophic lateral sclerosis and frontotemporal dementia. *The Lancet Neurology*.

[40] Kouri N, Whitwell JL, Josephs KA, Rademakers R, Dickson DW (2011). Corticobasal degeneration: a pathologically distinct 4R tauopathy. *Nature Reviews Neurology*.

[41] Jellinger KA (2007). The enigma of vascular cognitive disorder and vascular dementia. *Acta Neuropathologica*.

[42] Weiner MW, Veitch DP, Aisen PS (2012). The Alzheimer’s disease neuroimaging initiative: a review of papers published since its inception. *Alzheimer’s and Dementia*.

[43] Perrin RJ, Fagan AM, Holtzman DM (2009). Multimodal techniques for diagnosis and prognosis of Alzheimer’s disease. *Nature*.

[44] Hampel H, Frank R, Broich K (2010). Biomarkers for Alzheimer’s disease: academic, industry and regulatory perspectives. *Nature Reviews Drug Discovery*.

[45] Matsuda H, Imabayashi E (2012). Molecular neuroimaging in Alzheimer’s disease. *Neuroimaging Clinics of North America*.

[46] Ferreira LK, Busatto GF (2011). Neuroimaging in Alzheimer’s disease: current role in clinical practice and potential future applications. *Clinics*.

[47] Pouratian N, Sheth S, Bookheimer SY, Martin NA, Toga AW (2003). Applications and limitations of perfusion-dependent functional brain mapping for neurosurgical guidance. *Neurosurg Focus*.

[48] Schoonenboom NS, Reesink FE, Verwey NA (2012). Cerebrospinal fluid markers for differential dementia diagnosis in a large memory clinic cohort. *Neurology*.

[49] Buchhave P, Minthon L, Zetterberg H, Wallin AK, Blennow K, Hansson O (2012). Cerebrospinal fluid levels of *β*-amyloid 1–42, but not of tau, are fully changed already 5 to 10 years before the onset of Alzheimer dementia. *Archives of General Psychiatry*.

[50] Heister D, Brewer JB, Magda S, Blennow K, McEvoy LK (2011). Predicting MCI outcome with clinically available MRI and CSF biomarkers. *Neurology*.

[51] Vemuri P, Wiste HJ, Weigand SD (2010). Serial MRI and CSF biomarkers in normal aging, MCI, and AD. *Neurology*.

[52] Wright BL, Lai JT, Sinclair AJ Cerebrospinal fluid and lumbar puncture: a practical review.

[53] Mattsson N, Rosen E, Hansson O (2012). Age and diagnostic performance of Alzheimer disease CSF biomarkers. *Neurology*.

[54] Roher AE, Esh CL, Kokjohn TA (2009). Amyloid *β* peptides in human plasma and tissues and their significance for Alzheimer’s disease. *Alzheimer’s and Dementia*.

[55] Mayeux R, Schupf N (2011). Blood-based biomarkers for Alzheimer’s disease: plasma A*β*40 and A*β*42, and genetic variants. *Neurobiology of Aging*.

[56] Song F, Poljak A, Valenzuela M, Mayeux R, Smythe GA, Sachdev PS (2011). Meta-analysis of plasma amyloid-*β* levels in Alzheimer’s disease. *Journal of Alzheimer’s Disease*.

[57] Marcello A, Wirths O, Schneider-Axmann T, Degerman-Gunnarsson M, Lannfelt L, Bayer TA (2009). Circulating immune complexes of A*β* and IgM in plasma of patients with Alzheimer’s disease. *Journal of Neural Transmission*.

[58] Fleisher AS, Raman R, Siemers ER (2008). Phase 2 safety trial targeting amyloid *β* production with a *γ*-secretase inhibitor in Alzheimer disease. *Archives of Neurology*.

[59] Borroni B, Agosti C, Marcello E, Di Luca M, Padovani A (2010). Blood cell markers in Alzheimer disease: amyloid precursor protein form ratio in platelets. *Experimental Gerontology*.

[60] Noguchi-Shinohara M, Hamaguchi T, Nozaki I, Sakai K, Yamada M (2011). Serum tau protein as a marker for the diagnosis of Creutzfeldt-Jakob disease. *Journal of Neurology*.

[61] Bielewicz J, Kurzepa J, Czekajska-Chehab E, Stelmasiak Z, Bartosik-Psujek H (2011). Does serum tau protein predict the outcome of patients with ischemic stroke?. *Journal of Molecular Neuroscience*.

[62] Liliang PC, Liang CL, Weng HC (2010). Tau proteins in serum predict outcome after severe traumatic brain injury. *Journal of Surgical Research*.

[63] Blennow K, Hampel H, Weiner M, Zetterberg H (2010). Cerebrospinal fluid and plasma biomarkers in Alzheimer disease. *Nature Reviews Neurology*.

[64] Ray S, Britschgi M, Herbert C (2007). Classification and prediction of clinical Alzheimer’s diagnosis based on plasma signaling proteins. *Nature Medicine*.

[65] O'Bryant SE, Xiao G, Barber R (2011). A blood-based screening tool for Alzheimer’s disease that spans serum and plasma: findings from TARC and ADNI. *PLoS ONE*.

[66] Bjorkqvist M, Ohlsson M, Minthon L, Hansson O (2012). Evaluation of a previously suggested plasma biomarker panel to identify Alzheimer’s disease. *PLoS ONE*.

[67] Rosa GD, Salzano G, Caraglia M, Saccardi AA (2012). Nanotechnologies: a strategy to overcome blood-brain barrier. *Current Drug Metabolism*.

[68] Chalbot S, Zetterberg H, Blennow K (2011). Blood-cerebrospinal fluid barrier permeability in Alzheimer’s disease. *Journal of Alzheimer’s Disease*.

[69] Cai Z, Zhao B, Ratka A (2011). Oxidative stress and *β*-amyloid protein in Alzheimer’s disease. *NeuroMolecular Medicine*.

[70] Farias G, Cornejo A, Jimenez J, Guzman L, Maccioni RB (2011). Mechanisms of tau self-aggregation and neurotoxicity. *Current Alzheimer Research*.

[71] Park SY, Tournell C, Sinjoanu RC, Ferreira A (2007). Caspase-3- and calpain-mediated tau cleavage are differentially prevented by estrogen and testosterone in *β*-amyloid-treated hippocampal neurons. *Neuroscience*.

[72] Rametti A, Esclaire F, Yardin C, Terro F (2004). Linking alterations in tau phosphorylation and cleavage during neuronal apoptosis. *The Journal of Biological Chemistry*.

[73] Avila J (2010). Alzheimer disease: caspases first. *Nature Reviews Neurology*.

[74] de Calignon A, Fox LM, Pitstick R (2010). Caspase activation precedes and leads to tangles. *Nature*.

[75] Rissman RA, Poon WW, Blurton-Jones M (2004). Caspase-cleavage of tau is an early event in Alzheimer disease tangle pathology. *Journal of Clinical Investigation*.

[76] Basurto-Islas G, Luna-Muñoz J, Guillozet-Bongaarts AL, Binder LI, Mena R, Garcia-Sierra F (2008). Accumulation of aspartic acid421- and glutamic acid 391-cleaved tau in neurofibrillary tangles correlates with progression in Alzheimer disease. *Journal of Neuropathology and Experimental Neurology*.

[77] Reddy VP, Obrenovich ME, Atwood CS, Perry G, Smith MA (2002). Involvement of Maillard reactions in Alzheimer disease. *Neurotoxicity Research*.

[78] Garcia-Sierra F, Mondragon-Rodriguez S, Basurto-Islas G (2008). Truncation of tau protein and its pathological significance in Alzheimer’s disease. *Journal of Alzheimer’s Disease*.

[79] Hanger DP, Wray S (2010). Tau cleavage and tau aggregation in neurodegenerative disease. *Biochemical Society Transactions*.

[80] Henriksen K, Leeming DJ, Christiansen C, Karsdal MA (2011). Use of bone turnover markers in clinical osteoporosis assessment in women: current issues and future options. *Women’s Health*.

[81] Leeming DJ, Henriksen K, Byrjalsen I (2009). Is bone quality associated with collagen age?. *Osteoporosis International*.

[82] Henriksen K, Tanko LB, Qvist P, Delmas PD, Christiansen C, Karsdal MA (2007). Assessment of osteoclast number and function: application in the development of new and improved treatment modalities for bone diseases. *Osteoporosis International*.

[83] Rosenquist C, Fledelius C, Christgau S (1998). Serum CrossLaps One Step ELISA. First application of monoclonal antibodies for measurement in serum of bone-related degradation products from C-terminal telopeptides of type I collagen. *Clinical Chemistry*.

[84] Sassi ML, Eriksen H, Risteli L (2000). Immunochemical characterization of assay for carboxyterminal telopeptide of human type I collagen: loss of antigenicity by treatment with cathepsin K. *Bone*.

[85] Schaller S, Henriksen K, Hoegh-Andersen P (2005). In vitro, ex vivo, and in vivo methodological approaches for studying therapeutic targets of osteoporosis and degenerative joint diseases: how biomarkers can assist?. *Assay and Drug Development Technologies*.

[86] Karsdal MA, Henriksen K, Leeming DJ (2009). Biochemical markers and the FDA critical path: how biomarkers may contribute to the understanding of pathophysiology and provide unique and necessary tools for drug development. *Biomarkers*.

[87] Karsdal MA, Byrjalsen I, Henriksen K, Riis BJ, Christiansen C (2009). A pharmacokinetic and pharmacodynamic comparison of synthetic and recombinant oral salmon calcitonin. *Journal of Clinical Pharmacology*.

[88] Karsdal MA, Byrjalsen I, Azria M (2009). Influence of food intake on the bioavailability and efficacy of oral calcitonin. *British Journal of Clinical Pharmacology*.

[89] Karsdal MA, Byrjalsen I, Henriksen K, Riis BJ, Christiansen C (2010). Investigations of inter- and intraindividual relationships between exposure to oral salmon calcitonin and a surrogate marker of pharmacodynamic efficacy. *European Journal of Clinical Pharmacology*.

[90] Zinman B, Haffner SM, Herman WH (2010). Effect of rosiglitazone, metformin, and glyburide on bone biomarkers in patients with type 2 diabetes. *Journal of Clinical Endocrinology and Metabolism*.

[91] Aydin H, Mutlu N, Akbas NBG (2011). Treatment of a major depression episode suppresses markers of bone turnover in premenopausal women. *Journal of Psychiatric Research*.

[92] Geldmacher DS (2010). Alzheimer disease prevention: focus on cardiovascular risk, not amyloid?. *Cleveland Clinic Journal of Medicine*.

[93] Irie F, Fitzpatrick AL, Lopez OL (2008). Enhanced risk for Alzheimer disease in persons with type 2 diabetes and APOE *ε*4: the cardiovascular health study cognition study. *Archives of Neurology*.

[94] Brunholz S, Sisodia S, Lorenzo A, Deyts C, Kins S, Morfini G (2012). Axonal transport of APP and the spatial regulation of APP cleavage and function in neuronal cells. *Experimental Brain Research*.

[95] Grimm MO, Rothhaar TL, Hartmann T (2012). The role of APP proteolytic processing in lipid metabolism. *Experimental Brain Research*.

[96] Cookson MR (2009). *α*-Synuclein and neuronal cell death. *Molecular Neurodegeneration*.

[97] Eller M, Williams DR (2011). *α*-Synuclein in Parkinson disease and other neurodegenerative disorders. *Clinical Chemistry and Laboratory Medicine*.

[98] Zhang YJ, Xu YF, Dickey CA (2007). Progranulin mediates caspase-dependent cleavage of TAR DNA binding protein-43. *Journal of Neuroscience*.

[99] Rohn TT (2008). Caspase-cleaved TAR DNA-binding protein-43 is a major pathological finding in Alzheimer’s disease. *Brain Research*.

[100] Chen YS, Lim SC, Chen MH, Quinlan RA, Perng MD (2011). Alexander disease causing mutations in the C-terminal domain of GFAP are deleterious both to assembly and network formation with the potential to both activate caspase 3 and decrease cell viability. *Experimental Cell Research*.

